# Getting into the musical zone: trait emotional intelligence and amount of practice predict flow in pianists

**DOI:** 10.3389/fpsyg.2013.00853

**Published:** 2013-11-22

**Authors:** Manuela M. Marin, Joydeep Bhattacharya

**Affiliations:** ^1^Department of Basic Psychological Research and Research Methods, University of ViennaVienna, Austria; ^2^Department of Psychology, Goldsmiths, University of LondonLondon, UK

**Keywords:** optimal experience, altered states of consciousness, music performance, autotelic personality, emotion

## Abstract

Being “in flow” or “in the zone” is defined as an extremely focused state of consciousness which occurs during intense engagement in an activity. In general, flow has been linked to peak performances (high achievement) and feelings of intense pleasure and happiness. However, empirical research on flow in music performance is scarce, although it may offer novel insights into the question of why musicians engage in musical activities for extensive periods of time. Here, we focused on individual differences in a group of 76 piano performance students and assessed their flow experience in piano performance as well as their trait emotional intelligence. Multiple regression analysis revealed that flow was predicted by the amount of daily practice and trait emotional intelligence. Other background variables (gender, age, duration of piano training and age of first piano training) were not predictive. To predict high achievement in piano performance (i.e., winning a prize in a piano competition), a seven-predictor logistic regression model was fitted to the data, and we found that the odds of winning a prize in a piano competition were predicted by the amount of daily practice and the age at which piano training began. Interestingly, a positive relationship between flow and high achievement was not supported. Further, we explored the role of musical emotions and musical styles in the induction of flow by a self-developed questionnaire. Results suggest that besides individual differences among pianists, specific structural and compositional features of musical pieces and related emotional expressions may facilitate flow experiences. Altogether, these findings highlight the role of emotion in the experience of flow during music performance and call for further experiments addressing emotion in relation to the performer and the music alike.

## Introduction

Professional musicians often spend many months on practicing a musical piece, aiming at mastering its technical and interpretative challenges in order to prepare for a perfect performance in front of an audience. One possible explanation for performers' motivation to take on such intense musical practice on a daily basis for many years is the experience of flow (Csikszentmihalyi, 2002; Lamont, 2009; Custodero, 2012). Flow, or optimal experience, can be broadly defined as a psychological state involving the positive experience of being fully engaged in the successful pursuit of an activity (Csikszentmihalyi, 1990), and due to its intrinsically rewarding nature, flow seems to motivate humans to keep returning to the flow-inducing action and meeting greater challenges. Csikszentmihalyi (1990) developed a nine-dimensional flow construct. Based on these dimensions, flow is characterized by

challenge-skill balance (feeling competent enough to meet the high demands of the situation), action-awareness merging (doing things spontaneously and automatically without having to think), clear goals (having a strong sense of what one wants to do), unambiguous feedback (knowing how well one is doing during the performance itself), concentration on the task at hand (being completely focused on the task at hand), sense of control (having a feeling of total control over what one is doing), loss of self-consciousness (not worrying what others think of oneself), transformation of time (having the sense that time passes in a way that is different from normal), and autotelic experience (feeling the experience to be extremely rewarding). (Martin and Jackson, 2008, p. 146)

The construct of flow is conceptually similar to the construct of peak experience and peak performance, psychological states characterized by intense positive feelings and personal fulfillment (Maslow, 1968). These two constructs share many qualities with each other (Privette, 1983; Privette and Bundrick, 1991), and in fact, flow has been shown to be related to peak performances and high achievements across disciplines ranging from sports (e.g., Jackson, 1999), music performance (O'Neill, 1999; Sawyer, 2006; but also see Wrigley and Emmerson, 2013), to compositional creativity and meaningfulness (MacDonald et al., 2006; Baker and MacDonald, 2013). Here, we investigate individual differences among pianists with regard to flow experiences and their relationship to trait emotional intelligence and peak performance, respectively.

Most activities can be flow-inducing, irrespective of whether they are work or leisure-based (Csikzentmihalyi and Csikzentmihalyi, 1988). While there are considerable variations in the flow-inducing tasks and contextual settings, the flow experience itself is surprisingly similar across a range of demographic variables like culture, ethnicity, socioeconomic background and age (Csikzentmihalyi and Csikzentmihalyi, 1988; Clarke and Haworth, 1994; Moneta, 2004; Asakawa, 2010). However, large individual differences do exist in the characteristics of flow experience like its frequency and strength. Csikszentmihalyi (1990) has already proposed that certain personality traits, such as curiosity, persistence and low self-centeredness, may be characteristics of people who can easily achieve flow states. These personality traits may constitute what is known as an “autotelic personality” (Nakamura and Csikszentmihalyi, 2009). Autotelic persons look for more challenges (Logan, 1988), are less anxious, more motivated, and show higher ‘playfulness’ (Tan and Chou, 2010). However, we must point out here that not much is known about what constitutes an autotelic personality (Busch et al., 2013), and in fact, its existence is yet to be supported by substantial empirical evidence.

The limited number of studies on individual differences and dispositional flow vary in terms of types of individual differences and activities under investigation. For instance, the balance between skills and demands required by an activity only induced flow in those individuals that were characterized by an internal locus of control (Keller and Blomann, 2008; Mosing et al., 2012b). Locus of control is a personality construct (Rotter, 1966) that refers to people's beliefs regarding the action-outcome relationship. An internal locus of control is characterized by the belief that outcomes depend on controllable factors, such as attitude, preparation and effort, whereas an external locus of control is reflected in the belief that an outcome depends on the environment, luck and knowing the right people (Rotter, 1966; Levenson, 1981; Lefcourt, 1991). Since associations with happiness (e.g., Larson, 1989; DeNeve and Cooper, 1998) as well as with positive mental health (Naditch et al., 1975; Presson and Benassi, 1996) have been reported in individuals with a high internal locus of control, the finding by Keller and Blomann (2008) suggests that the underlying mechanism explaining this relationship may be rooted in varying degrees of sensitivity to the level of control during the pursuit of an activity. Individuals high in internal locus of control may enjoy the activity more when facing challenges and thus enter into flow states more easily.

Need for achievement was also identified as a personal characteristic that fosters flow experience through challenge-skill balance (Eisenberger et al., 2005). More generally, positive relationships between flow and mental toughness in sports (Crust and Swann, 2013), with personality traits reflecting a high need to learn and low need for activity in videogaming (Seger and Potts, 2012), as well as with self-control (Kuhnle et al., 2012), novelty seeking and persistence (Teng, 2011) were reported, respectively. A negative relationship between flow proneness and neuroticism was found with regard to activities in everyday life (Ullen et al., 2012). Furthermore, flow proneness and intelligence were not associated in a study involving adult twin pairs (Ullen et al., 2012).

Given the widely-studied relationship between flow, motivation, high achievement and individual differences in sports (for a review see Swann et al., 2012) and work-related activities (e.g., Csikszentmihalyi and LeFevre, 1989; Eisenberger et al., 2005; Nakamura and Csikszentmihalyi, 2009), it is surprising to note that the investigation of flow in music performance and composition has received comparatively little attention since Csikszentmihalyi's introduction of the flow concept (1975). One of the first studies on flow with regard to music was conducted by O'Neill (1999), who investigated the development of performance skills in adolescent musicians and their relation to flow by using the Experience Sampling Method. She found a positive relationship between high achievement in music performance and the number of experienced flow states. Custodero (2005) provided evidence for the existence of flow-like states in infants and children by investigating different musical learning environments. Moreover, MacDonald et al. (2006) revealed a positive relationship between creativity, flow and the quality of group compositions in university students. In a similar vein, the degree of flow experiences has been found to be positively correlated with the meaningfulness of songs created during therapeutic songwriting (Baker and MacDonald, 2013). In a longitudinal study, Fullagar et al. (2013) showed that high degrees of flow were accompanied with low experiences of performance anxiety in music performance students. The emergence of flow has also been examined in the context of choir singing and conducting (Custodero, 2002; Bloom and Skutnick-Henley, 2005; Freer, 2009) as well as in ensemble playing (Kraus, 2003; Sawyer, 2006).

More recently, researchers began to explore the psychophysiological underpinnings of flow states in pianists, revealing that flow is associated with decreased heart period, blood pressure and heart rate variability as well as with increased activity of the zygomaticus major muscle and respiratory depth (De Manzano et al., 2010). Thomson and Jaque (2011) investigated psychophysiological responses during flow states in performing musicians and found decreased cardiac autonomic balance and regulatory capacity.

Flow can also be experienced during music listening. For instance, Lamont (2009) discussed flow in the context of students' self-reported peak experiences in music listening and performance, exploring the ways in which music can lead to happiness. Similarly, Diaz (2011) was interested in investigating flow and its relation to mindfulness in the context of music listening and found that there may be a phenomenological difference between flow and aesthetic response. Flow was also associated with music listening in sports and exercise in elite-athletes (Laukka and Quick, 2011). Other recent studies addressed the issue of how to assess flow experiences in musicians (Martin and Jackson, 2008; Sinnamon et al., 2012; Wrigley and Emmerson, 2013), suggesting that flow scales developed for fields other than music, such as the flow scales by Jackson and Marsh (1996) and by Jackson and Eklund (2002), are reliable tools applicable to the domain of music. Taken together, research on musical flow offers valuable insights into questions relevant for psychologists, teachers, therapists and performers/composers alike.

Research on musicians' personalities has largely focused on differences between musicians of various instrument groups and musical styles (e.g., Buttsworth and Smith, 1995; Kemp, 1996; Cribb and Gregory, 1999; Langendörfer, 2008; Hernandez et al., 2009; Vuust et al., 2010), the relationship between personality and performance anxiety (e.g., Cooper and Wills, 1989; Marchant-Haycox and Wilson, 1992; Kenny et al., 2004), as well as the relationship between personality and creativity (e.g., Gibson et al., 2009; Charyton and Snelbecker, 2010). To our knowledge, studies focusing primarily on general aspects of personality (i.e., not music-specific traits such as performance anxiety) and their relation to flow experiences in musicians have not been conducted so far. Specifically, we explored whether there is something inherent in the personality of pianists (see e.g., Chmurzynska, 2012) that could help understand why some pianists reach flow states more often and easily compared to others.

Wrigley and Emmerson (2013) found in their study that flow experiences may depend on the family of specific instrument(s). Their results indicated that piano players reported lower levels of flow on average compared to brass and string players. Due to these differences of flow experiences observed in different instrument groups, we consider it as appropriate to focus our research on pianists. Furthermore, the piano is a common instrument in Western populations and also widely studied within the field of music performance research.

Flow is usually related to peak performances and subsequent happiness, and is as such a highly emotional experience. However, in music research, only a few studies have addressed flow theory in relation to emotion, which is rather surprising because emotions play a crucial role in musical communication (Juslin and Sloboda, 2010), possibly constituting an essential difference between the domains of sports and music (Sinnamon et al., 2012). For example, Bakker (2005) found support for the cross-over of flow between music teachers and their students by building on emotional contagion theory (Hatfield et al., 1994). The degree of flow experienced in teachers was correlated with the teachers' intrinsic work motivation, which was positively associated with the degree of flow experienced among their students.

Fritz and Avsec (2007) reported that positive emotional aspects of subjective well-being and dispositional aspects of flow were positively correlated in music students, whereas flow and satisfaction with life were less strongly correlated. The authors thus concluded that flow is more related to affective than cognitive aspects of subjective well-being. However, a great deal remains unknown about flow and its relation to emotion in music performance research. It can be surmised that the experience of flow during musical activities may depend on at least two “affective factors”: first, on the musical piece and its emotional characteristics, and second, on the performer's personality and his or her emotional intelligence. Both of these aspects were investigated in the current study by using self-report measures.

Emotional intelligence can be generally defined as “[…] the ability to process emotion-laden information competently and to use it to guide cognitive activities like problem solving and to focus energy on required behaviors” (Salovey et al., 2009). However, it is now standard in the field to differentiate between two constructs, namely trait emotional intelligence and ability emotional intelligence (for a review see Petrides, 2011). Trait emotional intelligence is measured via self-report and conceptualized as a personality trait, whereas ability emotional intelligence refers to emotion-related cognitive abilities and is measured via maximum-performance tests. Since the present study focuses on flow theory and emotion, trait emotional intelligence was chosen as a possible facet of a pianist's personality that may be associated with flow. This approach was also motivated by the fact that a positive relationship between trait emotional intelligence and length of musical training was reported earlier for a group of music students (Petrides et al., 2006). The primary goal of our study was to investigate whether trait emotional intelligence could predict dispositional flow in pianists. We hypothesized that a higher degree of trait emotional intelligence would be associated with a higher degree of dispositional flow.

Since a relationship between flow and superior performances and achievement was previously found by others (O'Neill, 1999; MacDonald et al., 2006; Baker and MacDonald, 2013), another goal of our study concerned the modeling of high achievement in piano performance as measured by having won prizes at piano competitions. It seemed plausible to assume that the experience of flow in piano performance would predict success in piano competitions. Finally, a set of self-developed questions explored whether there are specific characteristics of a musical piece that may induce flow states more easily than others. In particular, exploratory questions referred to emotions expressed and induced by the music, the musical style since emotional communication is associated with certain musical styles more than with others (Kallinen, 2005; Robinson, 2005), and also to the compositional style. If emotional intelligence and dispositional flow were related in the performer, these additional questions would help better understand the underlying mechanisms between flow and emotion in music performance and initiate future experiments.

## Methods and materials

### Participants

Participants were 76 piano performance students (including 45 females) who, at the time of this study, were pursuing a professional career as a musician. Seventy-three students were enrolled in a classical performance degree and three in a Jazz performance degree at English-speaking institutions of higher education (university or music conservatory). Fifty-six students were undergraduates. The participants had the following nationalities: UK (*n* = 29), US (*n* = 24), Australia (*n* = 17) and Canada (*n* = 6). The mean age was 21.7 years (*SD* = 3.7). Our participants started their piano training on average at 6.8 years (*SD* = 2.8) of age, played the piano as a first instrument for 14.0 years (*SD* = 5.0), and practiced on average 3.3 h a day (*SD* = 2.1) at the time of the study. Forty-five participants had previously won at least one prize in a piano competition. Thirty-seven participants indicated that they preferred to play the piano alone rather than together with others. Participants also estimated to improvise on average 1.8 h per week (*SD* = 3.2) on the piano. Twenty-one students reported regularly playing other instruments besides the piano. Our participants were thus considered to have a high degree of musical training and involvement with music at the time of the study.

### Materials

The questionnaire comprised two standardized tests, one on dispositional flow and one on trait emotional intelligence, as well as two self-developed questionnaires, one on flow and musical characteristics referring to emotion and musical style and one on the socio-demographic and musical backgrounds (musical training, musical preference, amount of practice etc.). The order of the administration of these separate questionnaires remained the same across all participants and was as follows: socio-demographic and musical background, flow scale, self-developed questions on flow and musical characteristics, and trait emotional intelligence scale.

The Dispositional Flow Scale-2 (DFS-2) (Jackson and Eklund, 2004) comprises 36 items referring to the nine-dimensional nature of flow and has been reliably applied (Cronbach alpha = 0.92) to assess flow in music performance (Sinnamon et al., 2012). Answers are collected on 5-point scales (1 = never to 5 = always) and require specific instructions depending on the activity under investigation. They were as follows: “Please answer the following questions in relation to your experience of practicing/playing a piano solo piece that you know by heart and which could be performed in a concert next week.” These concrete instructions were chosen to make participants think of a common and realistic situation of their lives as musicians and to enhance the comparability across their responses. Moreover, earlier research suggested that flow occurs more often at the last stages of practicing a new musical piece (Kraus, 2003). Furthermore, these instructions were considered as appropriate since we also aimed at investigating flow and peak performance.

The short form of the Trait Emotional Intelligence Questionnaire (TEIQue-SF) (Petrides and Furnham, 2006) measures global trait emotional intelligence by collecting responses to 30 items on 7-point scales (1 = completely disagree to 7 = completely agree).

The self-developed questionnaire on flow in the context of musical emotions and musical style involved questions about (i) flow and piano performance, (ii) flow and musical emotions, and (iii) flow and musical styles and composers. Depending on the type of question, answers involved yes/no responses, numeric or verbal responses, or responses on rating scales (either ranging from “yes, agree,” “yes, somehow agree,” “no, somehow disagree,” “no, don't agree” and “don't agree,” or from “always,” “frequently,” “sometimes,” “rarely,” “never” to “don't know”). The questions were developed by the first author, a musicologist, but also discussed with two professional pianists in order to ensure that all questions were meaningful to musicians and comprehensive. Participants were allowed to skip questions if they preferred not to respond to some of these questions, which was rarely the case.

Specifically, six questions assessed the number of flow states during piano performance and music listening (e.g., Would you agree that flow states in piano performance can only be reached when the piece is nearly ready for a public performance?), the relationship between flow and motivation (Would you agree that the experience of flow keeps you motivated to practice the piano and to become better?), flow and life-satisfaction (Do you experience a high degree of life satisfaction after the experience of flow in piano performance?) as well as the occurrence of flow by defining flow according to Csikszentmihalyi's concept (1990) prior to these questions: “Flow refers to an altered state of consciousness where one becomes so deeply immersed in a task that all else seems to disappear. This state is characterized by total concentration on the task at hand, clear goals, and unselfconscious action. Self-reports of flow include a transformation of our perception of time and self-awareness as well as a sense of fulfillment and feelings of intense happiness after a flow performance, referring to the intrinsically rewarding experience that flow brings to the individual.”

Several questions addressed the possible relationship between musical emotions and flow. Two questions probed the relationship between happiness and flow (Do you experience intense happiness and enjoyment WHILE being in a flow state in piano performance? Do you experience intense happiness and enjoyment shortly AFTER the experience of flow in piano performance?), two the general role of musical emotions in flow induction (Musical pieces are expressive of different types of emotions. From your own experience, do you feel that flow states are more easily induced by certain types of emotions expressed by a piece? Musical pieces can induce different types of emotions in you. From your own experience, do you feel that flow states are more easily induced when you feel certain types of emotions while playing a piece?), and two further questions referred to changing emotions in a musical piece (Would you agree that flow states appear less frequently when the emotional content of a piece is varying a lot over the course of a piece?) and general liking for certain emotions and their effect on flow states (Do you feel that flow states are more easily reached when the piece induces emotions in you that you particularly like in general (can be either positive or negative emotions?).

Two questions asked for specific ratings of flow in the context of musical emotions following Russell's circumplex model of affect (1980). The instructions ensured that participants understood the difference between felt and perceived emotions, a crucial distinction with regard to the study of musical emotions (Gabrielsson, 2002). The questions regarding flow in the context of Russell's circumplex model of affect were posed as follows: 1) “Emotions can be described by arousal (calm vs. activated) and pleasantness (pleasant vs. unpleasant). From your experience, please rate how often one of the following emotional states EXPRESSED by a piece has led to flow in piano performance. Note: You did not necessarily feel these emotions yourself while playing a piece,” followed by the specific emotions “low-arousing pleasant” “high-arousing pleasant,” “low-arousing unpleasant” and “high-arousing unpleasant” and the respective rating scale. The second question referred to felt emotions: “From your experience, please rate how often one of the following emotional states INDUCED by a piece has led to flow in piano performance. Note: These following emotions were not necessarily expressed by a piece, but they describe your feelings while playing a piece,” again followed by the specific emotions “low-arousing pleasant,” “high-arousing pleasant,” “low-arousing unpleasant” and “high-arousing unpleasant” and the respective rating scales.

The last five questions probed whether there was an association between musical styles, musical emotions and flow during piano performance. For example, participants were asked whether they had experienced flow states more often with certain musical styles than with others (Do you feel that you experience flow states more often when playing certain musical styles?), and further, to indicate the musical style that has most frequently induced flow states. Questions regarding the familiarity with and preference of musical styles complemented this section. Finally, participants were asked whether they could name any composers whose pieces had reliably induced flow during piano practice over a long period of time.

### Procedure

Music departments and piano professors in the UK, United States, Canada and Australia were contacted via email by the first author and invited to distribute the link to the online questionnaire among their students. This method of recruitment was chosen in order to ensure that participants were in fact piano performance students coming from higher institutions. Answering all the questionnaires took around 34 min on average, and participants could choose to participate in a prize draw. The data was collected between February and November 2010. This study was approved by the local ethics committee of the Department of Psychology at Goldsmiths, University of London, and followed the guidelines of the Declaration of Helsinki.

### Statistical analysis

Statistical analyses were conducted in IBM SPSS Statistics version 19 (SPSS Inc., Chicago, IL, USA) and in Matlab R2010b (The MathWorks, Inc., Natick, Massachusetts, USA). In order to control for type 1 error, we report adjusted *p*-values calculated for the non-parametric correlation analysis following the sequential Bonferroni-Holm procedure (Holm, 1979). This procedure is a sequentially rejective version of the simple Bonferroni correction for multiple comparisons, which strongly controls the family-wise error rate at level alpha. Howell (2002) recommends the Bonferroni-Holm procedure for multiple testing of several correlations from the same matrix. For regression analyses, it was ensured that all assumptions (no multicollinearity between the predictors, independence, homoscedasticity and normality of the errors) were met. Mediation regression analyses were computed using the SPSS macro “PROCESS” (Hayes, 2012). All statistical tests were two-tailed at an alpha level of 0.05 if not otherwise indicated.

## Results

### Relationship between trait emotional intelligence and flow experience

Three univariate outliers with 2 *SD* above or below the mean score were removed in the averaged DFS-2 scores and in the averaged TEIQue-SF scores, respectively. Reliability analyses (Cronbach, 1951) were conducted for each standardized scale after the removal of the outliers. For the DFS-2, Cronbach's alpha coefficient was calculated on all 36 items and yielded a value of α = 0.89 (*N* = 73). Individual analyses for each of the nine flow subscales revealed similarly high values between α = 0.71 for the subscale of action-awareness merging and α = 0.90 for the subscale of clear goals. Note that these values exceed Nunnally's (1978) criterion of 0.70 for acceptable reliability. Internal consistency was also assessed for the TEIQue-SF and considered as sufficiently high with a value of α = 0.83 (*N* = 73). Basic descriptives of the flow and trait emotional intelligence scales are displayed in Table 1.

**Table 1:** **Descriptive statistics of the DFS-2 scores, its nine subscales, and of TEIQue-SF (*N* = 73)**.

	***M***	***SD***	**Min**	**Max**	**α**
Mean flow score	3.37	0.38	2.67	4.25	0.89
Challenge-skill balance	3.53	0.60	2.50	5.00	0.80
Merging of action and awareness	3.21	0.60	2.00	5.00	0.71
Clear goals	3.74	0.79	1.75	5.00	0.90
Unambiguous feedback	3.50	0.65	1.50	5.00	0.84
Total concentration	3.24	0.58	2.00	5.00	0.77
Sense of control	3.14	0.52	2.00	5.00	0.74
Loss of self-consciousness	2.78	0.84	1.00	5.00	0.86
Transformation of time	3.50	0.78	1.75	5.00	0.81
Autotelic experience	3.66	0.80	2.00	5.00	0.87
Mean traitEI score	4.83	0.60	3.57	5.87	0.83

mean (M), standard deviation (SD), minimum (Min), maximum (Max), and Cronbach's alpha (α).

A comparison with the mean DFS-2 scores for each subscale as reported in Sinnamon et al. (2012) shows that, similar to their results reported for an sample of elite music performance students (*N* = 80), the mean rating for the subscale of Loss of Self-consciousness was the lowest among the nine subscales. In fact, the current result of 2.78 is similar to the reported mean value of 2.64 by Sinnamon et al. (2012), suggesting that Loss of Self-consciousness is a dimension of flow that may not be so relevant for flow in music performance. In the Sinnamon et al. (2012) study, the ranking of the nine subscales for the elite sample (studying music performance on a full-time basis) showed that Clear Goals (4.28), Autotelic Experience (4.19), Clear Feedback (3.96) and Challenge-skill Balance (3.92) were the four dimensions with the highest mean ratings. In our sample of piano performance students, Clear Goals (3.74), Autotelic Experience (3.66), Challenge-skill Balance (3.53) and Transformation of Time (3.50) were the dimensions with the highest mean ratings, indicating an overlap of three out of four dimensions between these two studies involving music performance students.

As a next step, the relationships between the individual flow subscales and the global flow score were investigated by correlation analyses. Shapiro-Wilk normality tests indicated significant deviations from normality for six out of the nine subscales after the removal of outliers 2 *SD* above or below the mean. Therefore, non-parametric Spearman-Rho correlations (*r*_s_) were computed on the unaltered original scores (Table 2). The findings suggest that all nine subscales were moderately to highly correlated with the average global flow score. The subscale of Autotelic Experience was most highly correlated with global flow [*r*_s(74)_ = 0.80], followed by Sense of Control [*r*_s(74)_ = 0.72], Challenge-skill Balance [*r*_s(74)_ = 0.70] and Total Concentration [*r*_s(74)_ = 0.68]. The subscales of Transformation of Time [*r*_s(74)_ = 0.46], Loss of Self-consciousness [*r*_s(74)_ = 0.43], and Unambiguous Feedback [*r*_s(74)_ = 0.47] showed only moderate correlations with the average flow score. These results are reflected by the generally low inter-correlations between these subscales with all other subscales, suggesting that not all dimensions contributed equally strongly to the overall flow scores in pianists.

**Table 2:** **Spearman-Rho correlations between the global mean DFS-2 score and the mean scores of the nine flow subscales (*N* = 76)**.

**Measure**	**Challenge-skill balance**	**Merging of action and awareness**	**Clear goals**	**Unambiguous feedback**	**Total concentration**	**Sense of control**	**Loss of self-conscious-ness**	**Trans-formation of time**	**Autotelic experience**
Merging of action and awareness	0.22								
Clear goals	0.51^*^	0.12							
Unambiguous feedback	0.35	0.09	0.41^*^						
Total concentration	0.45^*^	0.23	0.39^*^	0.37					
Sense of control	0.50^*^	0.29	0.45^*^	0.37	0.56^*^				
Loss of self-consciousness	0.09	0.20	0.01	0.10	0.30	0.22			
Transformation of time	0.15	0.52^*^	0.13	−0.15	0.17	0.20	0.01		
Autotelic experience	0.63^*^	0.43^*^	0.37	0.26	0.49^*^	0.56^*^	0.32	0.24	
Mean flow score	0.70^*^	0.53^*^	0.59^*^	0.47^*^	0.68^*^	0.72^*^	0.43^*^	0.46^*^	0.80^*^

* p < 0.05 after Bonferroni-Holm correction; all dfs = 74.

To predict overall flow experience during piano playing, a multiple stepwise linear regression analysis was conducted with the following predictors: *traitEI* (trait emotional intelligence), *practice* (daily amount of piano practice), *training* (overall duration of piano training), *age piano* (age of first piano lesson), *age* and *gender* (males = 1, females = 2). The average DFS-2 score, *flow*, was entered as the dependent variable. Outliers with 2 *SD* above and below the mean were removed from all variables prior to the analysis and cases were deleted list-wise; this resulted in 62 participants for the regression analysis. Correlations between the predictors are shown in Table 3 and regression coefficients in Table 4. The basic descriptive values were as follows (*N* = 62): *traitEI* (*M* = 4.81, *SD* = 0.61), *practice* (*M* = 3.05 h, *SD* = 1.49), *training* (*M* = 13.90 years, *SD* = 3.53), *age piano* (*M* = 6.35 years, *SD* = 2.00), *age* (*M* = 21.00 years, *SD* = 2.5), *gender* (23 males, 39 females), *flow* (*M* = 3.34, *SD* = 0.38).

**Table 3:** **Pearson product-moment correlations between the average flow score and six predictors (*N* = 62)**.

**Measure**	**Gender**	**Age**	**Practice**	**Training**	**Age piano**	**TraitEI**
Age	−0.18					
Practice	−0.12	0.25				
Training	−0.03	0.49	0.12			
Age piano	−0.07	−0.12	−0.07	−0.70		
TraitEI	0.03	0.09	0.44	0.05	0.02	
Flow	−0.10	0.08	0.48	0.11	−0.05	0.45

Practice, daily amount of piano practice; training, overall duration of piano training; age piano, age of first piano lesson; traitEI, mean TEIQue-SF score; flow, mean DFS-2 score.

**Table 4:** **Summary of stepwise regression analysis for six variables predicting flow in piano performance students (*N* = 62)**.

**Variable**	***B***	***SE B***	**β**
**STEP 1**			
Constant	2.97	0.10	
Practice	0.12	0.03	0.48^***^
Adjusted *R*^2^	0.22		
*F*	17.92^***^		
**STEP 2**			
Constant	2.2	0.34	
Practice	0.09	0.03	0.35^**^
TraitEI	0.18	0.08	0.29^*^
Adjusted *R*^2^	0.27		
*F*	12.47^***^		
Δ *R*^2^	0.07		

* p < 0.05

** p < 0.01

*** p < 0.001; B, non-standardized regression coefficient; β, standardized regression coefficient, SE, standard error; practice, daily amount of piano practice in hours; traitEI, mean TEIQue-SF score.

**Figure 1:**
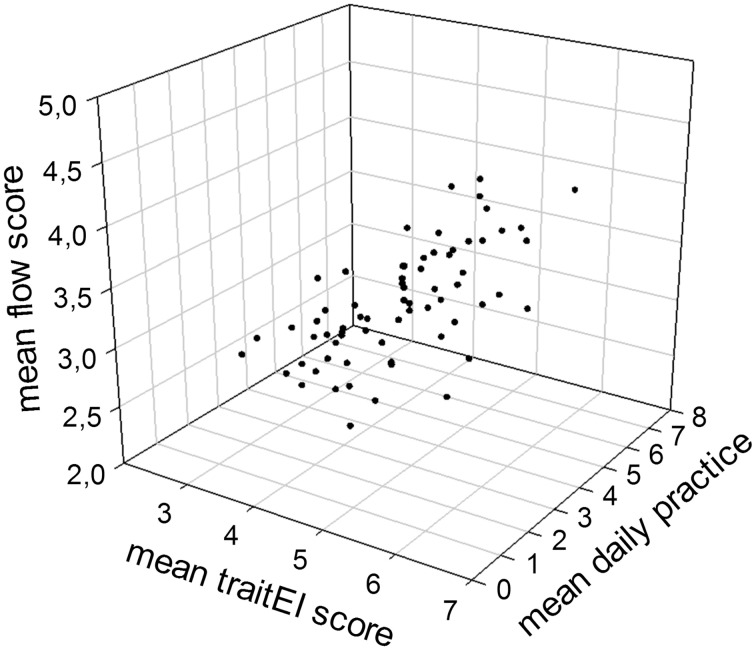
**Relationship between mean trait emotional intelligence scores, average amount of daily practice and mean dispositional flow scores in piano performance students (*N* = 62)**.

After two steps, the model was found to be successful in predicting flow experiences, *F*_(2, 59)_ = 12.47, *p* < 0.001. Two predictors, namely daily amount of practice and trait emotional intelligence, explained 27.0% of the overall variability of the flow scores (adjusted *R*^2^ = 0.27). The sizes and significances of β-values indicated that daily amount of practice was the most important predictor, but that trait emotional intelligence contributed significantly to an improvement of the model in a second step, β = 0.29, *t*_(55)_ = 2.37, *p* = 0.021. In other words, the results were in line with our hypothesis that trait emotional intelligence and flow experience are positively correlated. The positive linear association between amount of practice, trait emotional intelligence and flow is depicted in Figure 1.

Next we explored the underlying relationships between trait emotional intelligence, the amount of daily practice and flow. This analysis was motivated by previous research showing that musical training is correlated with emotional intelligence (Petrides et al., 2006) and the recognition of emotional prosody in speech (Lima and Castro, 2011), with even some causal evidence for an effect of musical training (Thompson et al., 2004), and that musicians respond differently to musical emotions compared to non-musicians (Dellacherie et al., 2011; Marin et al., 2012). We decided to test a mediation model based on a bootstrapping approach (Hayes, 2012) with amount of daily practice as the independent variable (*X*), flow experience as the dependent variable (*Y*) and emotional intelligence as a mediator (*ME*). The analysis was conducted by the SPSS macro PROCESS (Hayes, 2012). First, a mediator model was computed, a dependent variable model was computed in a second step, and finally a confidence interval for the indirect effect was computed applying a bias-corrected resampling bootstrap technique with 5000 resamples. All relationships between the variables were modeled as linear and visually inspected prior to the analysis.

Table 5 summarizes the results of the mediation regression analysis. The model predicting trait emotional intelligence was significant, *F*_(1, 66)_ = 13.70, *p* < 0.001, indicating that daily practice and trait emotional intelligence were positively correlated with each other. The model predicting flow experience was also significant, *F*_(2, 65)_ = 13.43, *p* < 0.001, explaining around 30% of the variance in flow experience. We observed a significant direct effect of daily practice on flow experience, indicating how much a unit change in practice affects flow experience independent of its effect on trait emotional intelligence. Furthermore, there was a positive correlation between emotional intelligence and flow experience, after controlling for daily practice. Last, we found a significant positive indirect effect, implying that an increase of daily practice led to an increase in flow experience through the effect of daily practice on emotional intelligence. The mediation effect size (*R*^2^) was small (0.12); therefore, these results should be interpreted with caution. For example, exchanging the independent variable with the mediator and vice versa did not change the overall results of the model. An alternative path model assuming that an increase in flow experiences increases levels of emotional intelligence through the effect of flow on practice was also tested and revealed a similar pattern of results. Since the inter-correlations between the three variables were similar in direction and strength, it was difficult to assess which model may be the correct one. At present, concrete theories on the relationship between flow, emotion and practice are lacking and thus cannot guide mediation analysis. Therefore, the current results should be regarded as exploratory.

**Table 5:** **Indirect effect of daily amount of practice on flow experience through trait emotional intelligence (*N* = 68)**.

	**Model predicting traitEI (ME)**		
	***Coeff***	***SE***	
Constant	4.28	0.15	
Practice (X)	0.17^***^	0.05	
Summary of model predicting ME	*R*^2^ = 0.17^***^		
	**Model predicting flow experience (Y)**		
Constant	2.09^***^	0.32	
Practice (X)	0.08^*^	0.03	
TraitEI (ME)	0.21^**^	0.07	
Summary of model predicting Y	*R*^2^ = 0.29^***^		
	**Indirect effect**	**CI 95%**	
	0.04	0.01	0.07

X, independent variable, ME, mediator variable, Y, dependent variable, Coeff, coefficient, CI, confidence interval.

* p < 0.05

** p < 0.01

*** p < 0.001.

### Relationship between flow and high achievement

Another testable hypothesis of interest concerned the relationship between flow and high achievement in piano performance. Therefore, to predict the likelihood that a piano student has won a prize in a piano competition (as a measure of high achievement), a binary logistic regression model (stepwise forward using the likelihood ratio statistic) was fitted to the data with seven predictors: *traitEI*, *practice*, *training*, *gender*, *age*, *age piano* and *flow*. Non-prize winners (*N* = 24) were coded as 1 and prize winners (*N* = 38) as 2. All assumptions for this statistical analysis were also verified. A test of the final model after two steps vs. a model with intercept only was statistically significant, χ^2^(2) = 17.09, *p* < 0.001. The model was able to correctly classify 73.7% of those who won a prize and 54.2% of those who did not, for an overall success rate of 66.1%. Table 6 shows the logistic regression coefficient, Wald test and odds ratio for each of the two significant predictors of the model, namely *practice* and *age piano*. The odds ratio of *practice* indicates that for each one hour increase in piano practice per day, there is a doubling of the odds that the piano performance student would win a prize, when other variables are controlled. In other words, the odds increase around 111% for a change of 1 h of practice. This interpretation of the result is reliable because the respective 95% confidence interval was >1 (lower boundary = 1.28, upper boundary = 3.47). The second significant predictor in the model was negative and referred to the age at which piano performance students began their piano training. The odds ratio was 0.69 and the respective 95% confidence interval was <1 (lower boundary = 0.50, upper boundary = 0.97), meaning that the odds of winning a prize in a competition were 0.69 lower for those who started their piano training one year later, or that there is a 31% decrease in the odds for winning a prize for each one-year increase in the age at which piano training began. For interpretational purposes one can also invert the odds ratio for this negative predictor, which shows that, for each one-year decrease in the age at which piano training began, the odds of winning a prize in a piano competition increase by a multiplicative factor of 1.44 (44%). In summary, although amount of practice and the age at which the lessons began were shown to be significant predictors, our hypothesis that flow experiences predicts high achievement in piano performance was not corroborated by the current data.

**Table 6:** **Regression coefficients and overall model evaluation for a logistic regression analysis using seven predictors to model high achievement in piano performance (*N* = 62)**.

	**Wald's**					***e*^β^**
**Predictor**	***B***	***SE B***	***X*^2^**	***df***	***p***	**(odds ratio)**
**STEP 1**						
Constant	−1.63	0.72	5.12	1	0.024^*^	NA
Practice	0.74	0.25	8.72	1	0.003^**^	2.09
**STEP 2**						
Constant	0.67	1.24	0.29	1	0.589	NA
Practice	0.75	0.25	8.63	1	0.003^**^	2.11
Age piano	−0.37	0.17	4.54	1	0.033^*^	0.69
**Test**			***X*^2^**	***df***	***p***	
**STEP 1**						
Overall model evaluation			11.93	1	0.001^**^	
Score test						
Goodness-of-fit test			6.86	6	0.334	
Hosmer and Lemeshow						
**STEP 2**						
Overall model evaluation			17.09	2	<0.001^***^	
Score test						
Goodness-of-fit test			11.82	8	0.160	
Hosmer and Lemeshow						

NA = non-applicable; Step 1: Cox and Snell R^2^ = 0.18, Nagelkerke R^2^ = 0.24; Step 2: Cox and Snell R^2^ = 0.24, Nagelkerke R^2^ = 0.33;

* p < 0.05

** p < 0.01

*** p < 0.001.

### Flow, musical emotions and musical styles

The analysis of the self-developed questionnaire on flow and emotion in music performance showed that 69 out of 76 pianists had experienced flow as defined by the nine-dimensional concept of flow. Sixty-three pianists estimated to experience on average 5.00 flow states (*SD* = 6.37) per month during piano playing and 7.56 flow states per month (*SD* = 8.97) during music listening. A Wilcoxon signed-rank test (*N* = 56) showed that the number of estimated flow states during music listening was significantly higher than the one during piano performance, *T* = 262, *p* = 0.004, *r* = −0.39. A Spearman-Rho correlation further indicated that there was a significant positive correlation between the number of flow states experienced during piano performance and music listening in a typical month, *r*_s(55)_ = 0.50, *p* < 0.001. The majority of participants also reported that flow experiences kept them motivated to practice the piano and to become better. The frequency of answers was as follows: 65.2% “yes, agree,” 20.3% “yes, somehow agree,” 5.8% “no, somehow disagree,” 1.5% “no, don't agree” and 7.2% of the pianists did not know. Moreover, the majority of pianists (*N* = 68) somehow or completely agreed that flow states in piano performance can only be reached when the piece is nearly ready for a public performance: 19.1% “yes, agree,” 48.5% “yes, somehow agree,” 14.7% “no, somehow disagree,” 11.8% “no, don't agree” and 5.9% of the pianists did not know. Last, we were also interested in whether flow and life satisfaction were linked in piano performance students and the data revealed the following answers (*N* = 66): 43.9% “always,” 30.3% “frequently,” 18.2% “sometimes,” 1.5% “rarely,” 0% “never” and 6.1% “don't know.”

Several items of the questionnaire referred to the relationship between flow and emotion in piano performance. Specifically, two questions addressed whether flow experiences are accompanied with intense feelings of happiness, differentiating between happiness *during* and *after* flow states piano performance. For the question referring to happiness during flow states, the pattern of results was as follows: 39.7% “always,” 20.6% “frequently,” 22.1% “sometimes,” 10.3% “rarely”, 2.9% “never” and 4.4% “don't know.” Happiness after flow states was even more common, as shown by the following replies: 46.4% “always,” 30.4% “frequently,” 13.0% “sometimes,” 2.9% “rarely,” and 7.3% “don't know.”

Next, a set of items referred to musical emotions, that is, emotions that are expressed or induced by the musical structure, and 62 out of 69 participants who experienced flow during piano performance agreed that flow states are more easily induced by certain types of emotions expressed by a musical piece than by others. In a similar vein, 61 out of 69 participants also responded that flow states depend on the nature of emotions induced by music. Table 7 summarizes responses to how often emotions varying in arousal and pleasantness, which were either expressed or induced by the music, led to flow in the current sample of pianists. For both expressed and induced musical emotions, low-arousing unpleasant emotions were not so frequently associated with flow states than other emotions, such as high-arousing pleasant and unpleasant emotions.

**Table 7:** **Emotions varying in arousal and pleasantness and their frequency of being related to flow states**.

**Musical emotions**	**Very often**	**Often**	**Sometimes**	**Never**
**EXPRESSED EMOTIONS (*N* = 66)**				
Low-arousing pleasant	11	15	39	1
High-arousing pleasant	18	25	22	1
Low-arousing unpleasant	8	13	33	12
High-arousing unpleasant	13	19	28	6
**INDUCED EMOTIONS (*N* = 65)**				
Low-arousing pleasant	9	26	28	2
High-arousing pleasant	16	28	19	2
Low-arousing unpleasant	5	17	36	7
high-arousing unpleasant	13	16	31	5

Two other questions addressed emotion and flow. First, participants (*N* = 69) were asked to indicate whether they would agree that flow states appear less frequently when the emotional content of a piece varies a lot over the course of a piece. Thirty-six point two per cent of the pianists answered “no, somehow disagree,” 17.4% with “no, don't agree,” 24.6% with “yes, somehow agree,” 11.6% with “yes, agree” and 10.1% with “don't know,” which suggests the existence of two subgroups in our sample, those who agree (36.2%) and those who do not (53.6%). Second, participants (*N* = 69) indicated whether they felt that flow states are more easily reached when the piece induces emotions that they particularly like in general (can be either positive or negative emotions). Here, the pattern of results was more clear and showed that the majority of pianists agreed (44.9%) or somehow agreed (37.7%), whereas only 13.0% somehow disagreed and 4.35% did not know. Taken together, these exploratory results are in line with the view that flow is a highly emotional experience, and further, suggest that musical emotions may play an important role in the induction of flow in performing artists.

A final set of items probed whether musical emotions and flow experience were associated through the musical style during piano performance. Participants (*N* = 67) reported whether they experienced flow more often when playing certain musical styles. Most participants agreed that the musical style played a role in flow states. The frequency of answers was as follows: 35.8% “yes, agree,” 35.8% “yes, somehow agree,” 10.5% “no, somehow disagree,” 7.5% “no, don't agree” and 10.5% of the pianists did not know. Furthermore, participants (*N* = 68) were asked to select the musical style in which they had experienced flow in piano performance most frequently. Pianists associated most frequently the Romantic style with flow (64.7%), followed by Classical (13.2%), Contemporary (8.8%), Baroque (2.9%), Other (10.3%) and Jazz (0%). In order to see whether this finding conforms to the pianists' preference for and familiarity with these musical styles, two other questions relating to the musical background were analyzed. First, a question referred to pianists' (*N* = 76) most favorite musical style in piano performance and the pattern of results was as follows: Romantic (57.9%), Contemporary (14.5%), Classical (10.5%), Baroque (6.6%), Jazz (5.3%) and Other (5.3%). Second, pianists (*N* = 76) had to indicate which musical style they played most frequently during the last five years: Romantic (42.1%), Classical (31.6%), Contemporary (11.8%), Baroque (4.0%), Jazz (2.6%) and Other (7.9%). In summary, for this specific sample of piano performance students, the Romantic style was the most familiar, preferred and also most flow-inducing. This finding corresponds to pianists' (*N* = 68) large agreement on the question whether flow states are more easily reached when playing pieces that they particularly like: 69.1% “yes, agree,” 25.0% “yes, somehow agree,” 2.9% “no, somehow disagree,” 1.5% “no, don't agree” and 1.5% of the pianists did not know.

Next we analyzed the possible link between familiarity, preference and flow induction with regard to musical styles at an individual level (Table 8). For thirty-one participants (45.6%) out of 68, the most frequently played musical style was also the most flow-inductive style, regardless of the type of musical style. The results further indicated that a high number of pianists (*n* = 25) selected the Romantic style as the most frequently played and most flow-inductive. However, the data also showed that 14 pianists who frequently played the classical style chose the Romantic style as the most flow-inductive style, suggesting that the Romantic style may be more flow-inductive than other styles. Similarly, we assessed whether there was a relationship between preference for a musical style and frequent flow induction. For forty-two (61.8%) out of 68 participants, the most favorite musical style was also the most flow-inductive style, regardless of the type of musical style. Romantic music was the most preferred musical style and also the most flow-inductive style for 34 piano performance students. Ten participants who had not chosen Romantic music as the most favorite style indicated that Romantic music was frequently flow-inductive.

**Table 8:** **Relationships between the most frequent occurrence of flow and the frequency of playing a musical style and the preference for a musical style, respectively (*N* = 68)**.

**Most flow**	**Baroque**	**Classical**	**Romantic**	**Contemporary**	**Jazz**	**Other**
**MOST FREQUENTLY PLAYED MUSICAL STYLE**						
Baroque	0	1	0	1	0	0
Classical	0	3	3	2	1	0
Romantic	1	14	25	1	0	3
Contemporary	1	1	1	3	0	0
Jazz	0	0	0	0	0	0
Other	0	1	1	1	1	3
**MOST FAVORITE MUSICAL STYLE**						
Baroque	0	1	0	0	0	1
Classical	1	2	2	2	2	0
Romantic	1	3	34	5	1	0
Contemporary	1	0	2	3	0	0
Jazz	0	0	0	0	0	0
Other	0	0	3	0	1	3

**Figure 2:**
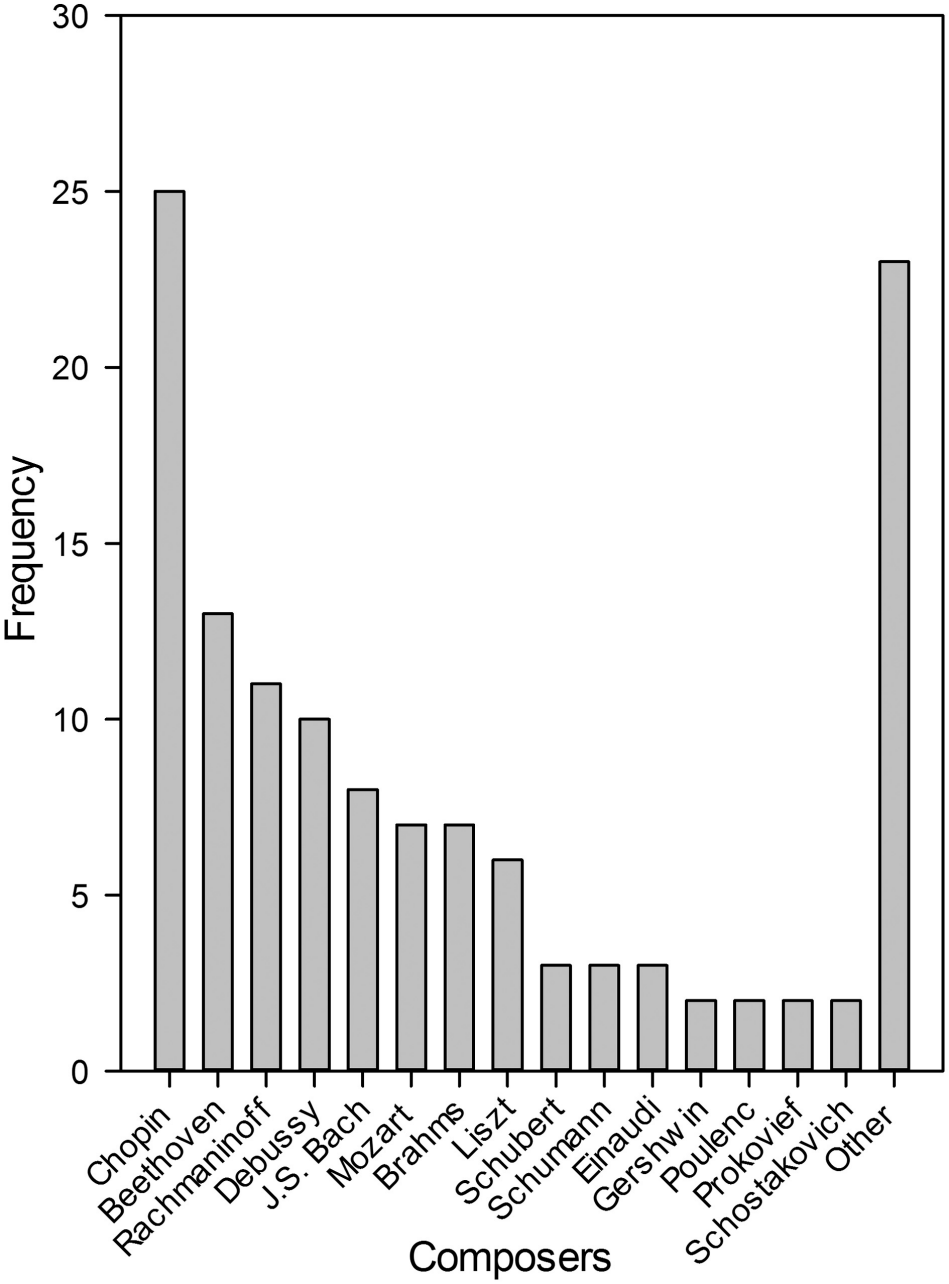
**Frequency distribution of composers who repeatedly induced flow states in a sample of piano performance students (*N* = 69)**. More than one name could be given.

Finally, we asked pianists (*N* = 69) to name composers whose pieces had reliably induced flow states in the past (different pieces by the same composer over a longer period of time). Pianists could name as many composers as they wished. Fifteen pianists did not name any composer. The responses of the other pianists were counted and those composers that were only named once were added to the category “Other.” Note that pianists could name more than one composer and all responses were considered in the analysis. Figure 2 depicts that Frédéric Chopin (1810–1849) was clearly the most often named composer, mentioned by 25 pianists, followed by Beethoven (13), Debussy (12) and J. S. Bach (8). These findings not only show that pianists were able to relate composers to flow experiences, but also that there was high agreement among pianists that Chopin's music is particularly flow-inducing.

## Discussion

Given the apparent paucity of research on flow and individual differences in music performance, the present study sought to investigate flow in relation to trait emotional intelligence in piano performance students. The rationale for this approach lies in the facts that being in a flow state is regarded as a highly emotional and intrinsically rewarding experience (Csikszentmihalyi, 1990), that music is strongly communicative of emotions (Juslin and Sloboda, 2010), and that being able to effectively deal with musical emotions may thus underlie the proneness of achieving a flow state during music performance. Further support for our approach to study flow in relation to emotion was recently provided by a study suggesting that the proneness to experience flow may be associated with personality dimensions that are under dopaminergic control and be reflected in low impulsiveness, stable emotion and positive affect (De Manzano et al., 2013). This is in line with findings by Montag et al. (2011), who observed that during listening to pleasant and unpleasant music individual differences with regard to self-transcendence modulate activity in the ventral striatum, which is part of the reward-circuitry. Last, recent research on the underlying genetic architecture of individual differences with respect to general flow proneness indicated that the same genetic factors may influence flow experienced across domains, whereas specific environmental factors may explain differences in flow proneness in different domains (Mosing et al., 2012a,b). Based on these findings we hypothesized that there is a positive association between trait emotional intelligence and flow experiences among musicians.

Our correlational study comprised a sample of undergraduate and postgraduate piano performance students (*N* = 76), implying that these students were professionally engaged with piano playing. We did not test for pianists' ability to deal with musical emotions but used a general personality test (Petrides and Furnham, 2006) to predict the disposition to achieve flow states (Jackson and Eklund, 2002) during piano performance. A stepwise regression analysis, including trait emotional intelligence, gender, age, age of first piano training, duration of piano training, and daily amount of piano practice as predictors, revealed that besides the amount of piano practice, trait emotional intelligence was the only other significant predictor in the model. In other words, the higher the trait emotional intelligence of a piano performance student, the more prone is s/he to experience flow. The positive association between the two variables is in line with models of emotional intelligence that claim that the ability to get into a flow state is a sign of high emotional intelligence (Goleman, 1995). It remains to be seen whether this positive relationship between trait emotional intelligence and dispositional flow can also be observed under experimental conditions and in domains outside music. Music performance may be a kind of activity in which emotional communication plays a larger role than in other physical and cognitive activities.

Our regression model demonstrated a positive relationship between trait emotional intelligence, daily amount of practice and flow, and yielded an adjusted *R*^2^ of around 0.27. Thus, it can be argued that the model needs to be extended and improved by including other predictors. Given that gender, age and predictors related to musical training were not predictive of flow in this rather homogeneous sample of piano performance students, it seems pertinent to assume that other personality features that are not covered by our study may also contribute to flow experience. Therefore, future models could include those traits that have been predictive of flow in domains outside music. For instance, locus of control (Keller and Blomann, 2008) as well as self-control (Kuhnle et al., 2012), novelty seeking and persistence (Teng, 2011) have been associated with flow experiences. Moreover, the investigation of mediation effects in a set of personality traits may be a promising avenue for future research on the existence of an autotelic personality among musicians.

The underlying relationship between daily amount of piano practice, trait emotional intelligence and dispositional flow was further examined by fitting a mediator model to the data. Research on the relationship between amount of musical training and emotional responses to musical emotions (Dellacherie et al., 2011; Marin et al., 2012 but see Bigand et al., 2005) and emotional prosody in speech (Thompson et al., 2004; Lima and Castro, 2011; Thompson et al., 2012; but see Trimmer and Cuddy, 2008) is somewhat relevant for the current research and was thus taken as a conceptual starting point for modeling effects of the amount of daily practice on flow through trait emotional intelligence. Our mediator model was significant, but similar results were also obtained for an alternative path model (flow—practice—emotional intelligence). This clearly limits the interpretation of the suggested mediator model and more (experimental) research is needed to elucidate the relationship between practice behavior, trait emotional intelligence and flow during music performance.

A set of self-developed questions corroborated the hypothesis that the ease of experiencing flow is related to the emotions expressed and induced by a musical piece. The majority of participants (around 89%) acknowledged the role of musical emotions in flow induction. The results further suggest that pleasant and unpleasant high-arousing musical emotions are more associated with the experience of flow than unpleasant low-arousing musical emotions, a finding which was valid for both expressed and induced musical emotions. Future experiments may explore the role of specific types of musical emotions and musicians' ability to deal with them with regard to the nine flow dimensions proposed by Csikszentmihalyi (1990). One testable hypothesis that directly follows from the current results is that high-arousing pleasant musical emotions may be more strongly associated with the dimension of autotelic experience because the latter is usually accompanied with enjoyment and happiness, which are both characterized by high arousal. In other words, it is possible that a congruency between musical emotions and emotions inherent to autotelic experience may facilitate the latter state. From our perspective, the current research could also be extended by adding tests on emotional ability involving musical stimuli, which may offer additional insight into unresolved questions regarding the role of emotions in flow induction.

Since it is known that musical styles vary in their degree of emotional expressivity (Kallinen, 2005), we also explored whether the degree of induced flow may depend on the musical style. Our data suggests that pianists largely support the view that flow experiences occur more often with certain musical styles (around 72% “agreed” or “somehow agreed” with this statement). The majority of our participants associated Romantic music, and particularly the music by Frédéric Chopin, with flow experiences. However, although the Romantic era and its music are generally regarded as being strongly expressive of emotions (Robinson, 2005), this musical style was also the most familiar and preferred one among pianists. Further analyses based on individual relationships between these variables revealed that for 45.6% of the participants the most familiar musical style was also the most flow-inductive style, regardless of the type of musical style. Nonetheless, 14 participants who most frequently played classical music associated the Romantic style with flow experience. Accordingly, there is mild evidence that familiarity may not be the sole explanation in the flow-musical style relationship. We further observed a link between the most favorite musical style and flow in 61.8% of the participants, regardless of the specific musical style. Of course, further studies would be necessary to disentangle effects of familiarity and preference on flow from those that are due to the musical structure of the style.

A binary logistic regression model was computed to predict high achievement among piano performance students (i.e., having won prizes at piano competitions). Our hypothesis that enhanced levels of experienced flow may predict high achievement in piano performance could not be supported by the current data. Instead, the logistic regression model indicated that the amount of daily practice and the age at which piano training began were the only significant predictors. This result is essentially in line with research on professional achievement in music performance which regards experience and practice as crucial for superior expert performance (Sloboda et al., 1996; Lehmann and Ericsson, 1997; Gabrielsson, 2003), but which also suggests that superior music performance may be a multifaceted phenomenon that is conceptually complex and difficult to model (Hallam, 1997; Ericsson, 2006). For instance, recent research has shown that visual information largely influences judgments of musical performances in competitions (Tsay, 2013), which may partly explain why flow did not predict high achievement in the current sample of pianists. A related issue concerns the possible link between high achievement and some external locus of control, which may counteract the positive relationship between internal locus of control, flow and high achievement. Our finding that the age of the first piano training was predictive of success in piano performance is in line with results indicating that there may be a sensitive period in early childhood where musical practice in the form of motor training may lead to benefits for performance in adulthood (e.g., Watanabe et al., 2007; Penhune, 2011).

A dissociation between high degrees of flow and high achievement has been previously reported in sports (Jackson, 1999). In a similar vein, Wrigley and Emmerson (2013) investigated flow states during a music performance examination and did not find that students who further progressed in their studies experienced flow more often than those who did not. Privette (1983) discussed the differences and similarities between the constructs of peak experience, peak performance and flow. She suggested that, for example, the notion of playfulness may be essential to flow but not to peak performance and further, that a strong sense of self is common for peak performances but not for flow. Although there is a substantial overlap between these constructs, differences on one dimension of the construct, in combination with effects of social context (e.g., practice vs. performance vs. exam), may explain discrepancies in research results and should thus be considered in future research.

The current study also provided some insights into the question of whether all nine dimensions of the flow concept developed by Csikszentmihalyi (1990) contribute equally well to the global flow score as assessed by the frequently used dispositional and state flow scales developed by Jackson and colleagues (Jackson and Marsh, 1996; Jackson and Eklund, 2002). In general, we found positive correlations between all nine subscales and the global DFS-2 scale, indicating that all different dimensions of dispositional flow play a role in flow experienced during music performance. Sinnamon et al. (2012), also assessing dispositional flow, reported that the DFS-2 subscales of Transformation of Time and Loss of Self-consciousness correlated more weakly with the other flow subscales in a sample of music students. It is interesting to note that whereas the current study asked piano performance students to think of performing a piece that is already well-mastered, Sinnamon et al. (2012) asked their participants to rate the items based on their experience of performing music in general. In both cases, the dimensions of Transformation of Time and Loss of Self-consciousness appeared as being less correlated with other flow dimensions, corroborating previous findings in the domain of sports (e.g., Jackson, 1996; Jackson et al., 2001; Jackson and Eklund, 2002). This finding may also indicate that the specific instructions of current studies on dispositional flow in music performance did not largely affect the inter-relationships between the subscales and the relationship with the global flow score. Moreover, our results suggest that the subscale of Unambiguous Feedback is another dimension of flow that is less correlated with other subscales, which is in line with reports by Sinnamon et al. (2012). Finally, previous research on musical flow involving the Flow State Scale-2 (Jackson and Marsh, 1996) reported that the subscale Transformation of Time was among the weakest predictors of global flow state and that Autotelic Experience, Sense of Control and Challenge-skill Balance were among the strongest (Wrigley and Emmerson, 2013). Our current results are similar to these findings. In summary, these results illustrate that the inter-relationships between the global flow scale and its subscales may be similar when flow is assessed in different scenarios of music performance (dispositional vs. state). However, more empirical evidence is needed to support this claim.

In conclusion, this study highlights the role of emotions in flow experience in two ways. First, individual differences regarding trait emotional intelligence predict dispositional flow, and second, pianists acknowledge the role of musical emotions in the induction of flow. Both findings warrant further experimental investigations for generalizations, by including other instrument groups and artistic activities, such as dancing and painting, in which emotional communication is also vital.

## Author contributions

Manuela M. Marin and Joydeep Bhattacharya conceived and designed the research. Manuela M. Marin collected and analyzed the data. Manuela M. Marin and Joydeep Bhattacharya wrote the article.

### Conflict of interest statement

The authors declare that the research was conducted in the absence of any commercial or financial relationships that could be construed as a potential conflict of interest.
